# PAM-Flexible Genome Editing with an Engineered Chimeric Cas9

**DOI:** 10.21203/rs.3.rs-2625838/v1

**Published:** 2023-03-07

**Authors:** Sabrina Koseki, Lauren Hong, Vivian Yudistyra, Teodora Stan, Emma Tysinger, Rachel Silverstein, Christian Kramme, Nadia Amrani, Natasha Savic, Martin Pacesa, Tomás Rodriguez, Manvitha Ponnapati, Joseph Jacobson, George Church, Ray Truant, Martin Jinek, Benjamin Kleinstiver, Erik Sontheimer, Pranam Chatterjee

**Affiliations:** Duke University; Duke University; Duke University; Duke University; MIT Media Lab; Massachusetts General Hospital; Harvard Medical School; RNA Therapeutics Institute, University of Massachusetts Medical Schoo; McMaster University; EPFL; UMass Medical School; MIT Media Lab; Massachusetts Institute of Technology; Harvard Medical School; McMaster University; University of Zurich; Massachusetts General Hospital and Harvard Medical School; University of Massachusetts Medical School; Duke University

## Abstract

CRISPR enzymes require a defined protospacer adjacent motif (PAM) flanking a guide RNA-programmed target site, limiting their sequence accessibility for robust genome editing applications. In this study, we recombine the PAM-interacting domain of SpRY, a broad-targeting Cas9 possessing an NRN > NYN PAM preference, with the N-terminus of Sc++, a Cas9 with simultaneously broad, efficient, and accurate NNG editing capabilities, to generate a chimeric enzyme with highly flexible PAM preference: SpRYc. We demonstrate that SpRYc leverages properties of both enzymes to specifically edit diverse NNN PAMs and disease-related loci for potential therapeutic applications. In total, the unique approaches to generate SpRYc, coupled with its robust flexibility, highlight the power of integrative protein design for Cas9 engineering and motivate downstream editing applications that require precise genomic positioning.

## Introduction

To conduct programmable genome editing, CRISPR-associated (Cas) endonucleases require a protospacer adjacent motif (PAM) to immediately follow the target DNA sequence specified by the guide RNA (gRNA).^1–3^ PAM binding triggers DNA strand separation, enabling base pairing between the gRNA and the target DNA strand for subsequent nucleolytic cleavage and editing events.^4,5^ The widely-utilized Cas9 from *Streptococcus pyogenes* bacteria (SpCas9), for example, requires a 5’-NGG-3’ PAM,^2,6,7^ imposing severe accessibility constraints for therapeutically-relevant editing applications requiring precise genomic positioning, such as base editing and homology-directed repair.^8–12^

To expand the targetable sequence space of CRISPR, we previously engineered Sc++, a variant of ScCas9 which employs a positive-charged loop that relaxes the base requirement at the second PAM position, thus enabling a 5’-NNG-3’ preference, rather than the canonical 5’-NGG-3’.^13,14^ Concurrent with the development of Sc++, Walton, *et al*. engineered a near-PAMless Cas9, termed SpRY, which contains mutations in the PAM-interacting domain (PID) of SpCas9 that enable strong 5’-NRN-3’ specificity, alongside weaker 5’-NYN-3’ targeting.^15^ Both Sc++ and SpRY thus represent exciting advances in CRISPR-based genome editing due to their robust editing characteristics and unprecedented genomic accessibility, respectively.^16^

In this study, we combine Sc++ and SpRY to engineer a chimeric Cas9 enzyme that can induce edits with highly orthogonal PAM targeting. To do this, we employ experimental enzyme engineering and computational modeling to graft the PID of SpRY to the N-terminus of Sc++, generating a chimeric SpRY-Sc++ Cas9 (herein referred to as **SpRYc**). We demonstrate that SpRYc integrates the loop structure of Sc++ and the PID mutations of SpRY to specifically edit various 5’-NNN-3’ PAM targets in human cells, enabling unique editing applications. Finally, we conduct homology modeling to gain insights into the protein-DNA interactions that may enable SpRYc’s PAM flexibility. In total, SpRYc’s demonstrated PAM flexibility offers numerous opportunities for broad-targeting genome editing applications and therapeutic translation.

## Results

### Engineering of SpRYc

SpRY harbors ten substitutions in the PID of SpCas9 (L1111R, D1135L, S1136W, G1218K, E1219Q, A1322R, R1333P, R1335Q, and T1337R) which help reduce its specificity from the canonical 5’-NGG-3’ to the more flexible 5’-NRN-3’ PAM.^15^ Alternatively, ScCas9 and Sc++ both employ positive-charged, flexible loop-like structures in their N-terminus (residues 367 to 376) that do not exist in SpCas9 or SpRY, and relax the need for the second PAM base, enabling more minimal 5’-NNG-3’ PAM preference rather than 5’-NGG-3’.^13,14^

**Figure 1.**
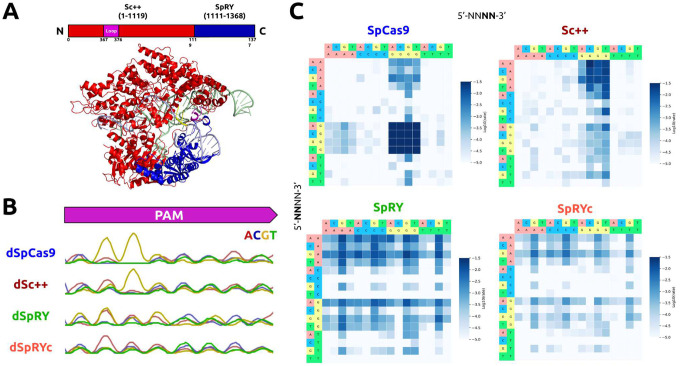
Engineering, Modeling, and PAM Characterization of SpRYc. (A) Homology model of SpRYc generated in SWISS-MODEL from PDB 4UN3 and visualized in PyMol. Original domain coordinates are indicated in parentheses above while SpRYc coordinates are indicated below. PAM is indicated in yellow, loop in purple, Sc++ N-terminus in red, and SpRY PID in blue. (B) PAM enrichment for indicated dCas9 enzymes utilizing PAM-SCANR. Each dCas9 plasmid was electroporated in duplicates, subjected to FACS analysis, and gated for GFP expression based on a negative “No Cas9” control and a positive dSpCas9 control. All samples were performed in independent transformation replicates, and the PAMs of the GFP-positive cells were sequenced via Sanger sequencing. (C) PAM profiles of SpCas9, Sc++, SpRY, and SpRYc proteins as determined by HT-PAMDA. Rate constants corresponding to Cas cleavage activity are illustrated as log10 values and are the mean of cleavage reactions against two unique spacer sequences.

Previously, we grafted the GC-independent PID of *Streptococcus macacae* Cas9 to the N-terminus of its ortholog, SpCas9, to generate iSpyMac, an efficient 5’-NAA-3’ editor.^17^ Motivated by our previous domain grafting results, we engineered a single variant possessing the critical properties of SpRY and Sc++ by rationally exchanging the PID of Sc++ with that of SpRY to generate a chimeric hybrid Cas9: SpRYc. SpRYc consists of the N-terminus (residues 1–1119) of Sc++, including the flexible loop, followed by the region of SpRY (residues 1111–1368) spanning its PID mutations (Figure 1A).

### PAM Characterization of SpRYc

To experimentally interrogate the PAM specificity of SpRYc in comparison to SpCas9, Sc++, and SpRY, we adapted a positive selection bacterial screen based on green fluorescent protein (GFP) expression conditioned on PAM binding, termed PAM-SCANR.^18^ Following transformation of the PAM-SCANR plasmid, harboring a PAM library, a single gRNA (sgRNA) plasmid targeting the fixed PAM-SCANR protospacer, and a corresponding nuclease-deficient dCas9 plasmid, we conducted FACS analysis to isolate GFP-positive cells in each population for subsequent library amplification and sequencing (Supplementary Figure 1). Our results suggest that while SpRY preferentially binds to an A or G at position 2 in the PAM, as expected, SpRYc more potently binds adenine bases at position 2, but does not bias against any specific base either (Figure 1B).

We performed additional experiments to assess PAM preference using HT-PAMDA, which calculates the cleavage rates of Cas9 enzymes (as opposed to an endpoint assay, like PAM-SCANR) on a library of substrates harboring different PAMs. While we observe far broader editing capabilities of SpRYc than the 5’-NGG-3’ PAM of SpCas9 and the strong 5’-NNG-3’ PAM of Sc++, SpRYc exhibited slower cleavage rates than SpRY, though able to access a comparably broad set of PAMs, thus suggesting that SpRYc may elicit its optimal activity in either its “dead” or nickase variants, rather than as a nuclease (Figure 1C). Overall, these results motivated us to evaluate SpRYc’s genome editing capability on endogenous loci in both nuclease and non-cleavage editing formats.

### Human Genome Editing Capabilities of SpRYc

**Figure 2.**
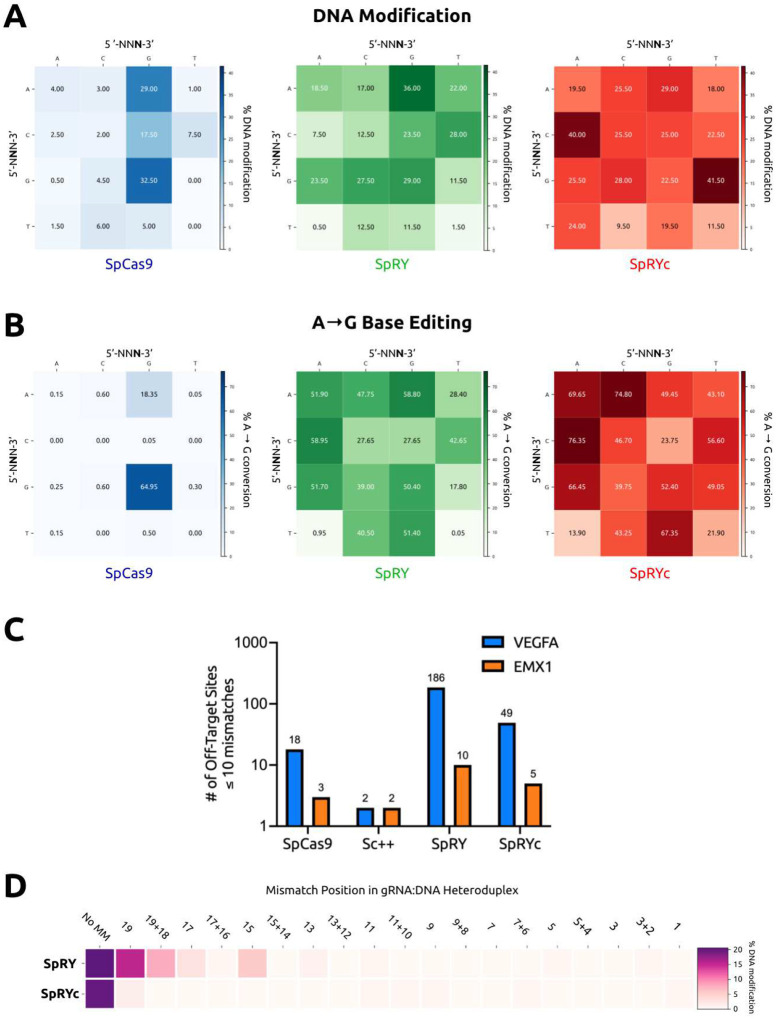
Broad, Efficient, and Specific Genome Editing Capabilities of SpRYc. (A) Quantitative analysis of indel formation with indicated Cas9 variants. Indel frequencies were determined via batch analysis following PCR amplification of indicated genomic loci, in comparison to unedited controls for each gene target. All samples were performed in independent transfection replicates and the mean of the quantified indel formation values was calculated. (B) Quantitative analysis of A-to-G with indicated ABE8e variants. Base editing conversion rates were determined via BEEP following PCR amplification of indicated genomic loci, in comparison to unedited controls for each gene target. All samples were performed in independent transfection replicates and the mean of the quantified base editing formation values was calculated. (C) Off-targets as identified by GUIDE-seq genome-wide for SpCas9, Sc++, SpRY, and SpRYc each paired with two sgRNAs targeting either *EMX1* or *VEGFA*. Only sites that harbored a sequence with ≤10 mismatches relative to the gRNA were considered potential off-target sites. (D) Efficiency heatmap of mismatch tolerance assay on genomic targets. Quantified indel frequencies are exhibited for each labeled single or double mismatch (number of bases 5’ upstream of the PAM) in the sgRNA sequence for the indicated Cas9 variant and indicated PAM sequence. All samples were performed in independent transfection replicates and the mean of the quantified indel formation values was calculated.

To evaluate SpRYc’s activity at diverse gene sequences, we compared the PAM specificities and DNA cleavage capabilities of SpRYc to SpCas9 and SpRY by transfecting HEK293T cells with plasmids expressing each Cas9 alongside one of sixteen sgRNAs which were directed to various genomic loci representing every two-base PAM combination (5’-N**NN**-3’) (Supplementary Table 1). Five days after transfection, indel formation was quantified following PCR amplification of the target genomic regions and subsequent sequencing analysis. Our results demonstrate that SpRYc generates modifications at all tested genomic loci, performing comparably to SpRY, and more optimally on select 5’-NYN-3’ loci. (Figure 2A). We similarly tested the performance of SpRYc in comparison to SpCas9 and SpRY for base editing applications by fusing each variant to ABE8e, a rapid, high-activity adenine base editor.^19,20^ We quantified editing efficiency of the base with the highest conversion percentage in the editing window following PCR amplification of the target genomic regions. Our results reveal that SpRYc-ABE8e can base edit at all tested genomic sequences, as compared to SpCas9-ABE8e and on certain, selected loci, more optimally than SpRY-ABE8e (Figure 2B). Taken together, our results suggest that SpRYc is able to target, cleave, and base edit at genomic sites with minimal dependence on a specific PAM.

### Reduced Off-Target Propensity of SpRYc

Previously, we demonstrated that Sc++ is an intrinsically high-fidelity enzyme, with far reduced off-targeting as compared to the standard SpCas9.^14^ We thus hypothesized that SpRYc may possess lower off-target propensity than its SpCas9-derived counterpart, SpRY. To investigate this hypothesis, we employed the genome-wide, unbiased GUIDE-Seq method,^21^ by utilizing sgRNA sequences targeting two previously analyzed genomic loci (*VEGFA* and *EMX1*). Our results demonstrate that compared to SpRY, SpRYc has nearly four-fold lower off-target activity with the VEGFA-targeting guide RNA, and two-fold lower off-target activity when directed against the EMX1 site (Figure 2C, Supplementary Figures 2–3). We corroborated this data via a mismatch tolerance assay,^22^ in which we employed sgRNAs harboring double or single mismatches to a fixed protospacer for an endogenous *DNMT1* locus. SpRYc exhibited decreased activity on mismatched sequences, as compared to SpRY, with slightly lowered on-target activity (Figure 2D). Our observations thus support our HT-PAMDA results that SpRYc’s attenuation in nuclease efficiency may result in fewer off-targets and improved mismatch tolerance.

### SpRYc Base Editors Mediate Therapeutically-Relevant Edits

**Figure 3.**
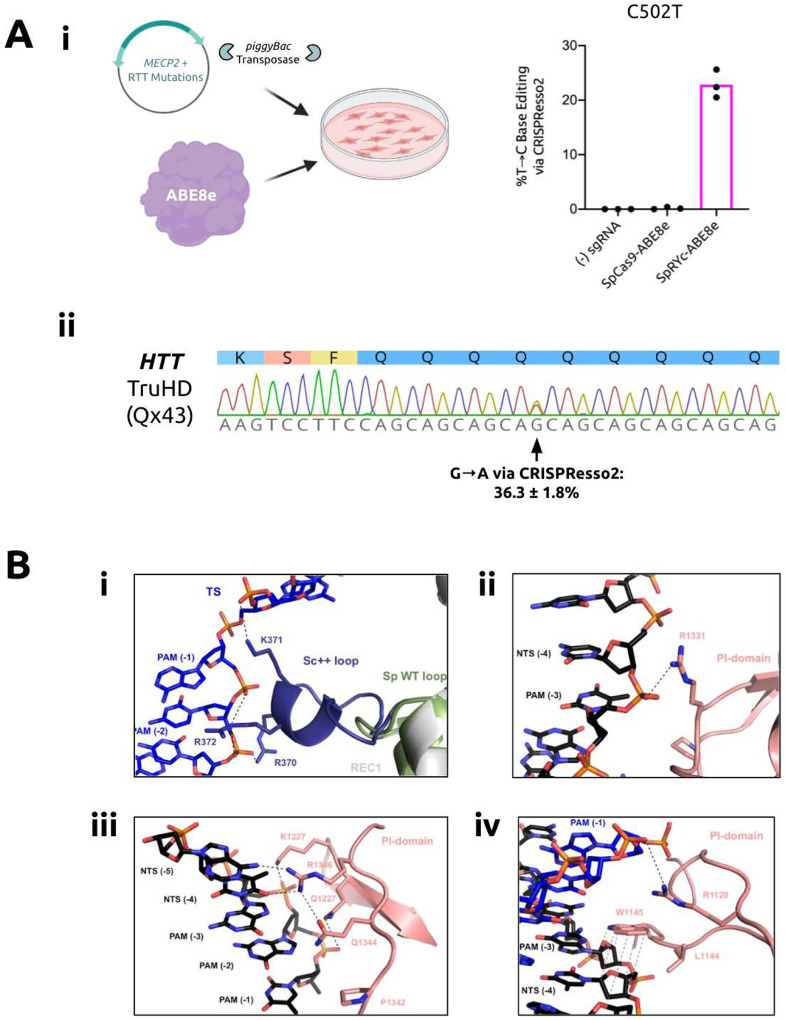
Potential applications and structural mechanisms of SpRYc. (A) Targeting disease-associated loci with SpRYc. (i) Schematic of SpRYc RTT Experiment. Base editing conversion rates were determined via CRISPResso2 NGS analysis following PCR amplification of *MECP2*-integrated loci, in comparison to unedited controls for the C502T installed mutation. Samples were performed in independent nucleofection triplicates (n=3) and the mean of the quantified base editing formation values was calculated. (ii) SpRYc-BE4Max was nucleofected into TruHD cells alongside an sgRNA targeting the *HTT* repeat. Base editing conversion rate was determined via CRISPResso2 NGS analysis NGS following PCR amplification of indicated genomic loci, in comparison to an unedited control. The analogous Sanger sequencing trace is shown. Samples were performed in independent nucleofection triplicates (n=3) and the mean of the quantified base editing formation values was calculated. (B) Structural insights via homology modeling in SWISS-MODEL. (i) Interaction of the engineered Sc++ loop (purple) with the backbone of the target strand (TS) PAM region. The REC1 loop from wild type SpCas9 is indicated in green. (ii) Potential interaction of residue R1331 with the non-target strand (NTS) backbone. (iii) Multiple mutations within the PAM interaction loop allow for a more flexible PAM readout. (iv) The potential van der Waals interaction of W1145 with the ribose moieties of non-target strand residues could further stabilize the PAM interaction.

Having established SpRYc’s relevant editing capabilities in human cells, we sought to investigate its utility as a potential therapeutic modality for the treatment of genetic diseases. Rett syndrome (RTT) is a progressive neurological disorder that predominantly affects young females. A majority of patients carry one of eight mutations in the *MECP2* gene (C316T, C397T, C473T, C502T, C763T, C808T, C880T, C916T), all of which are C-to-T substitution mutations and can thus be potentially ameliorated by CRISPR adenine base editors, such as ABE8e.^9,19,23^ Notably, one of the eight mutations, C502T, can only be accessed at target sites consisting of a 5’-NCN-3’ or 5’-NTN-3’ PAM, preventing its correction by previous adenine base editors. To test whether SpRYc-ABE8e can effectively correct the C502T mutation, we generated a universal RTT HEK293T cell line via *piggyBac* transposase-mediated integration of a synthetic gene fragment encoding *MECP2* installed with the aforementioned RTT mutations. After puromycin selection, we transfected the SpRYc-ABE8e plasmid alongside optimized sgRNAs for the C502T mutation site (Supplementary Table 1). After subsequent DNA extraction, loci amplification, and sequencing, we demonstrate that SpRYc-ABE8e can effectively mutate MECP2, including over 20% editing efficiency at the C502T mutation (Figure 3A).

Huntington’s Disease (HD) is a monogenic dominant neurological disorder affecting more than 1 in 10000 adults.^24^ It is caused by an expanded CAG repeat on chromosome 4 of the *HTT* gene, which encodes an extended polyglutamine (polyQ) tract in the resulting *huntingtin* protein.^24^ Recent studies have shown that there is an inverse relationship between the age of disease onset and the number of continuous CAG repeats, with significant benefit of a natural interrupting CAA codon on age onset and severity of disease.^25^ We therefore assessed SpRYc’s ability to introduce silent CAA interruptions in the CAG repeat region of HTT. To do this, we transfected patient-derived TruHD fibroblast cells, possessing a clinically-relevant CAG repeat length of 43 repeats.^26^ These lines are hTert immortalized, but not transformed and are very genomically stable. We used a cytosine base editor SpRYc-BE4Max alongside an sgRNA targeting the antisense strand of the HTT repeat region (Supplementary Table 1).^27^ Our NGS sequencing results show that SpRYc can install a CAA interruption at the fourth CAG repeat, with an editing efficiency of over 35%, thus shortening the uninterrupted repeat length by 4 and reducing the CAG tract length to the sub-pathogenic range (Figure 3B). Taken together, these results illustrate SpRYc’s potential utility for clinically-relevant applications and motivate its potential development as a therapeutic platform.

### *In silico* Modeling of SpRYc

To gain insights into the mechanisms of SpRYc’s PAM targeting, and owing to the nearly 90% sequence similarity between ScCas9 and SpCas9, we conducted homology modeling of SpRYc in the DNA substrate bound-state using the SWISS-MODEL server (Figure 1A and 3B).^28^ We hypothesized that the optimized loop of Sc++ may enforce targeting breadth by generating sequence-nonspecific interactions with the PAM to relax the need for an A or G at position 2. Homology models indicate that the engineered positively-charged loop inserted into the REC1 domain points towards the PAM region of the target DNA strands and thus potentially establishes new compensating interactions with the phosphate backbone of the target strand (Figure 3Bi). In addition, the combination of ScCas9 and SpRY mutations suggests several new non-specific backbone interactions with the non-target strand, thereby supporting a relaxed PAM profile of SpRYc (Figure 3Bii–iii). Of note is a potential van der Waals interaction of the aromatic side chain of W1145 with the ribose moieties of the proximal non-target strand residues (Figure 3Biv).^29^ These interactions, resulting from the engineered mutations, may thus energetically compensate for lack of PAM-specific recognition and facilitate local unwinding of double stranded DNA necessary for efficient R-loop formation in the absence of canonical PAM interactions.

## Discussion

While PAMs play a critical role in self-nonself discrimination by prokaryotic CRISPR-Cas9 immune systems, they limit the accessible sequence space for genome editing applications. Recent engineering and discovery efforts have yielded a host of Cas9 variants with altered or relaxed PAMs.^13–15,17,30–36^ In this study, we engineer a chimeric Cas9 by harnessing the structural properties of SpRY and Sc++ to generate SpRYc, a Cas9 with flexible PAM preference. While SpRYc did not demonstrate high cleavage rates in our HT-PAMDA assays, we do show that SpRYc has enhanced editing rates on diverse genomic loci as well as reduced off-target effects. SpRYc may be optimally fit for non-nuclease editing applications, including precise base editing, prime editing, and CRISPR-mediated activation or inhibition. Further, due to the high sequence homology of ScCas9 and SpCas9, we anticipate that high-fidelity mutations^22,37,38^ can easily be ported into SpRYc for improved specificity, as has been shown previously for both Sc++ and SpRY.^14,15^ Finally, we demonstrate that SpRYc can be integrated within base editing architectures to edit disease-related loci for potential therapeutic purposes.

While SpRYc serves as a step forward towards unrestricted, fully programmable genome editing, its development, more importantly, represents a culmination of a variety of state-of-the-art *in silico* and *in vitro* PAM engineering methods. ScCas9 was first identified via a high-throughput bioinformatics algorithm for ortholog discovery, dubbed SPAMALOT.^13^ Its derivative, Sc++, was engineered by computationally identifying and extracting motifs from *Streptococcus* orthologs, and splicing them into ScCas9 for improved functionality.^14^ Concurrently, SpRY was the result of a multi-year effort of SpCas9-based directed evolution and rational mutagenesis.^15,30^ Finally, a combination of structure-based homology modeling and domain grafting methods, those that were instrumental in engineering other PAM variants such as iSpyMac^17^ and cCas9,^36^ enabled the fusion of SpRY and Sc++ into our final SpRYc variant. Together, these studies emphasize the power of integrating diverse engineering modalities to generate new and useful proteins and open the door for future integrative protein design.

## Materials and Methods

### Generation of Plasmids

To generate SpRYc, the N-terminal ORF of Sc++ (Addgene Plasmid #155011), corresponding to residues (1–1119) was PCR amplified and assembled using Gibson Assembly into the pCMV-T7-SpRY-P2A-EGFP backbone (Addgene Plasmid #139989), preserving residues 1111–1368 of SpRY’s ORF. pCMV-T7-SpCas9-P2A-EGFP (Addgene Plasmid #139987) was used for SpCas9, and Sc++ was similarly integrated within the backbone. Analogously, the ORFs of SpCas9, SpRY, and SpRYc were integrated within the ABE8e (Addgene Plasmid #138489) and AncBE4Max (Addgene Plasmid #112094) backbones. sgRNA plasmids were constructed by annealing oligonucleotides coding for crRNA sequences (Table S1) as well as 4 bp overhangs, and subsequently performing a T4 DNA Ligase-mediated ligation reaction into a plasmid backbone immediately downstream of the human U6 promoter sequence. Assembled constructs were transformed into 50 μL NEB Turbo Competent *E. coli* cells, and plated onto LB agar supplemented with the appropriate antibiotic for subsequent sequence verification of colonies and plasmid purification.

### HT-PAMDA

We performed HT-PAMDA as described previously.^39^ Briefly, HEK 293T cells were transfected with plasmids encoding Cas9 nuclease variants, and *in vitro* cleavage assays were performed using the resulting cell lysates. sgRNAs were generated from Addgene plasmid #160136 with the T7 RiboMAX Express Large Scale RNA Production System (Promega). 180 ng of PAM library (Addgene #160132) was incubated with 30 nM of sgRNA and 6 μL of fluorescein-normalized lysate. PAM depletion was quantified following NGS of PCR-amplified undigested target DNA via the PAMDA software package: https://github.com/kleinstiverlab/HT-PAMDA. Cleavage rates for each Cas9 for each 5’-NNNN-3’ PAM can be accessed in Supplementary Table 2.

### PAM-SCANR Assay

Plasmids for the SpCas9 sgRNA and PAM-SCANR genetic circuit, as well as BW25113 ΔlacI cells, were generously provided by the Beisel Lab (North Carolina State University). Plasmid libraries containing the target sequence followed by either a fully-randomized 8-bp 5’-NNNNNNNN-3’ library or fixed PAM sequences were constructed by conducting site-directed mutagenesis, utilizing the KLD enzyme mix (NEB) after plasmid amplification, on the PAM-SCANR plasmid flanking the protospacer sequence (5’-CGAAAGGTTTTGCACTCGAC-3’). Nuclease-deficient mutations (D10A and H850A) were introduced to the ScCas9 variants using Gibson Assembly as previously described. The provided BW25113 cells were made electrocompetent using standard glycerol wash and resuspension protocols. The PAM library and sgRNA plasmids, with resistance to kanamycin (Kan) and carbenicillin (Crb) respectively, were co-electroporated into the electrocompetent cells at 2.4 kV, outgrown, and recovered in Kan+Crb Luria Broth (LB) media overnight. The outgrowth was diluted 1:100, grown to ABS600 of 0.6 in Kan+Crb LB liquid media, and made electrocompetent. Indicated dCas9 plasmids, with resistance to chloramphenicol (Chl), were electroporated in duplicates into the electrocompetent cells harboring both the PAM library and sgRNA plasmids, outgrown, and collected in 5 mL Kan+Crb+Chl LB media. Overnight cultures were diluted to an ABS600 of 0.01 and cultured to an OD600 of 0.2. Cultures were analyzed and sorted on a FACSAria machine (Becton Dickinson). Events were gated based on forward scatter and side scatter and fluorescence was measured in the FITC channel (488 nm laser for excitation, 530/30 filter for detection), with at least 10,000 gated events for data analysis. Sorted GFP-positive cells were grown to sufficient density, plasmids from the pre-sorted and sorted populations were isolated, and the region flanking the nucleotide library was then PCR amplified and submitted for Sanger sequencing or Amplicon-EZ NGS analysis (Genewiz). FCS files were analyzed using FCSalyzer https://sourceforge.net/projects/fcsalyzer/, and gating strategy is described in Supplementary Figure 1.

### Cell Culture and DNA Modification Analysis

HEK293T cells were maintained in DMEM supplemented with 100 units/ml penicillin, 100 mg/ml streptomycin, and 10% fetal bovine serum (FBS). sgRNA plasmids (100 ng) and nuclease plasmids (100 ng) were transfected into cells as duplicates (2 × 10^4^ / well in a 96-well plate) with Lipofectamine 3000 (Invitrogen) in Opti-MEM (Gibco). Five days after transfection, genomic DNA was extracted using QuickExtract Solution (Lucigen), and genomic loci were amplified by PCR utilizing the Phusion Hot Start Flex DNA Polymerase (NEB). Amplicons were enzymatically purified and submitted for Sanger sequencing or Amplicon-EZ NGS sequencing (Genewiz). Sanger sequencing ab1 files were analyzed using the ICE web tool for batch analysis (ice.synthego.com)^40^ in comparison to an unedited control to calculate indel frequencies via the ICE-D score. Select samples were further verified using the TIDE algorithm (tide.deskgen.com) to ascertain consistency of editing rates between replicates.^41^ NGS FASTQ files were analyzed using a batch version of the software CRISPResso2 (https://github.com/pinellolab/CRISPResso2).^42^ Base editing files were analyzed via the Based Editing Evaluation Program (BEEP) (https://github.com/mitmedialab/BEEP) in comparison to an unedited control. All samples were performed in independent duplicates or triplicates, as indicated.

### GUIDE-Seq

We performed GUIDE-Seq as described previously.^21^ Briefly, HEK293T cells were electroporated in a 24-well plate with 500 ng of Cas9, 500 ng of sgRNA, 10 ng of mCherry plasmids, and 7.5 pmol of annealed GUIDE-Seq oligonucleotide using the Neon nucleofection system (Thermo Fisher Scientific). After 72 hours post-nucleofection, genomic DNA was extracted with a DNeasy Blood and Tissue kit (Qiagen 69504) according to the manufacturer’s protocol. DNA libraries were prepared using custom oligonucleotides described in Tsai, *et al*.^21^ Library preparations were done with original adaptors with each library barcoded for pooled sequencing. The barcoded, purified libraries were sequenced on a MiniSeq platform in a paired-end (150/150) run.

Raw sequencer output (BCL) was demultiplexed and aligned to hg38 using GS-Preprocess (github.com/umasstr/GS-Preprocess).^43^ This software also constructed a reference of UMIs unique to each read and merged technical replicate BAM files. Off-target analysis of this input was performed using the GUIDEseq Bioconductor package.^44^ Only sites that harbored a sequence with ≤10 mismatches relative to the gRNA were considered potential off-target sites. GUIDE-Seq read count data is indicated in Supplementary Figures 2–3.

### Rett Syndrome Cell Line Generation

The *MECP2* editing locus containing all common Rett syndrome mutations was synthesized as a gBlock from IDT and inserted via Gateway cloning to a promoter-less *piggyBac* pMVP destination vector (Addgene #121874) harboring puromycin resistance. The RTT vector was then integrated into the HEK293T cell line via lipofection. Briefly, 600,000 cells were seeded in D10 media (DMEM + 10% FBS) to a six well plate 24 hours prior to lipofection. 2.5 ug of the RTT plasmid and 0.5 ug of a CMV-super PiggyBac transposase (System Biosciences) were then lipofected using Lipofectamine 3000 according to the manufacturer’s protocol. Media was changed six hours post-transfection and cells were subjected to 1 ug/ml puromycin selection 48 hours post-transfection for 3 days. Cells were then expanded under no drug selection for three days to allow non-integrated plasmid loss, then again selected for 3 additional days to isolate a pure population.

### TruHD Cell Culture

TruHD-Q43Q17M cells were cultured in MEM supplemented with 15% FBS and 1% Glutamax and grown under 4% O_2_ and 5% CO_2_ at 37°C in a 10 cm plate.^26^ At 95% confluence, cells were transfected through Lonza nucleofection using the SG Cell Line 4D-Nucleofector Kit. Growth media was replaced 24 hours post-nucleofection. 5 days post-nucleofection genomic DNA was extracted with PureLink Genomic DNA Mini Kit (Invitrogen).

### Statistical Analysis

Data are shown as the mean of all sample replicates. Data was plotted using Matplotlib and the Prism GraphPad software.

### Homology Modeling

Structural models of SpRYc were generated using the SWISS-MODEL server,^28^ using the PDB 4UN3 DNA substrate bound Cas9 model as template.^6^ Modeled sidechains and loops were curated and adjusted manually using COOT software.^45^

## Data Availability

All data needed to evaluate the conclusions in the paper are present in the paper and supplementary tables. All source data and sequencing files can be found at https://tinyurl.com/yxm2wpfx. SpRYc plasmids will be made available on Addgene.
